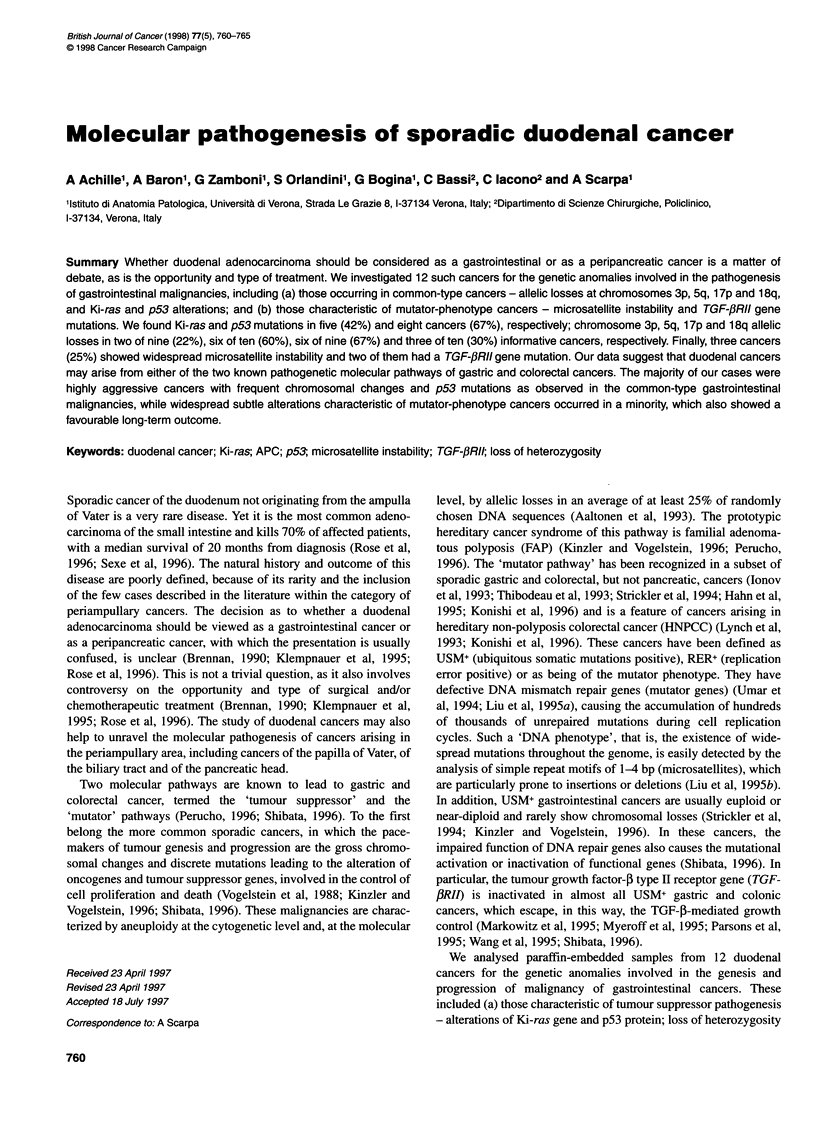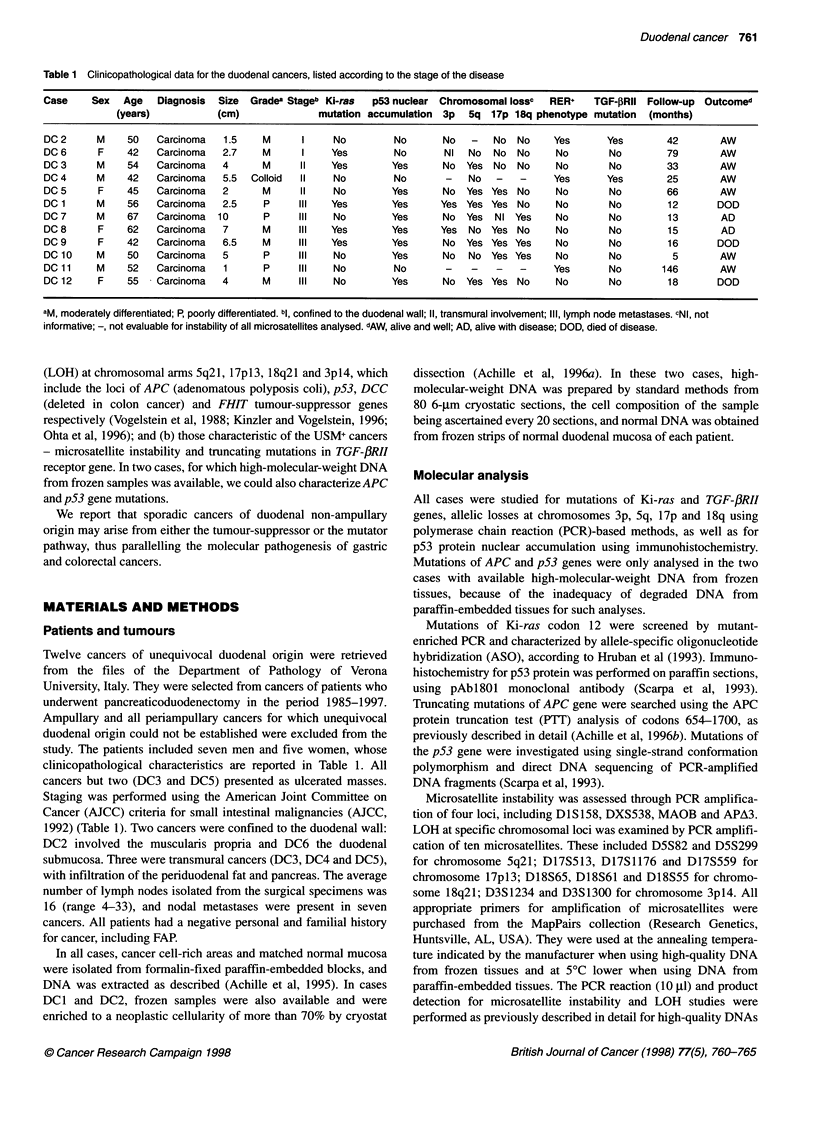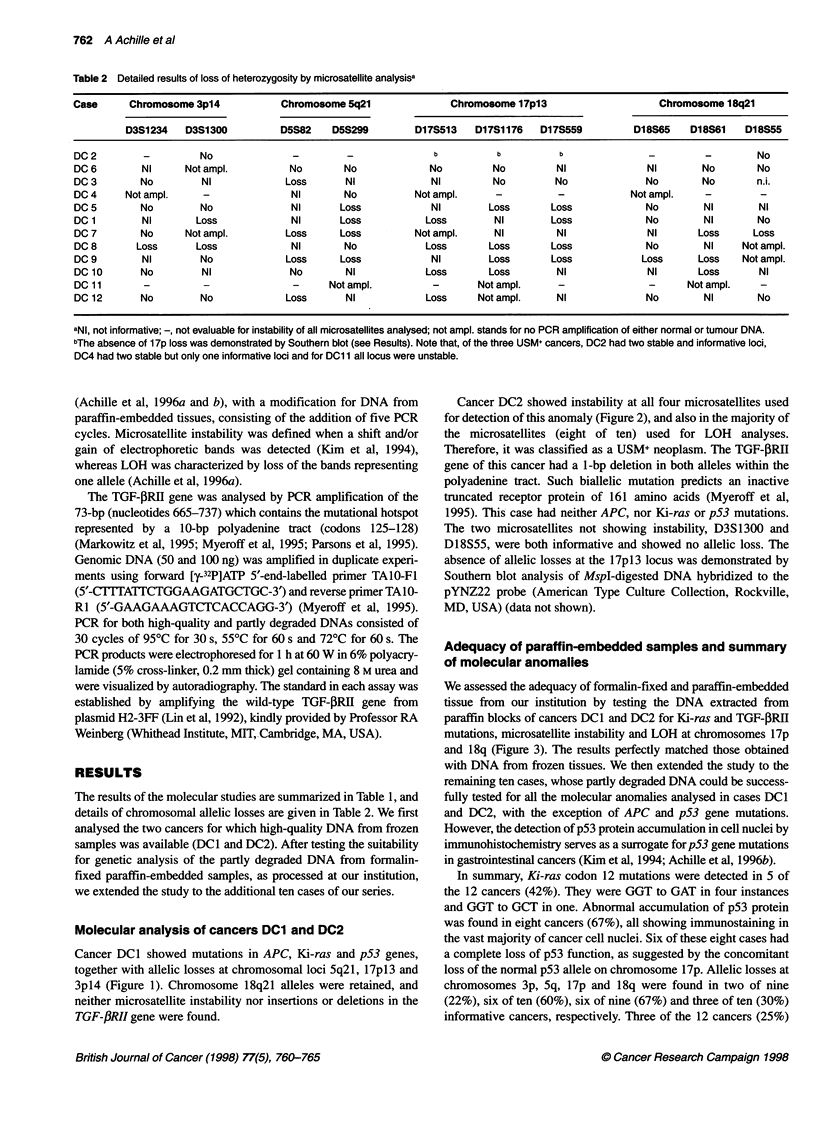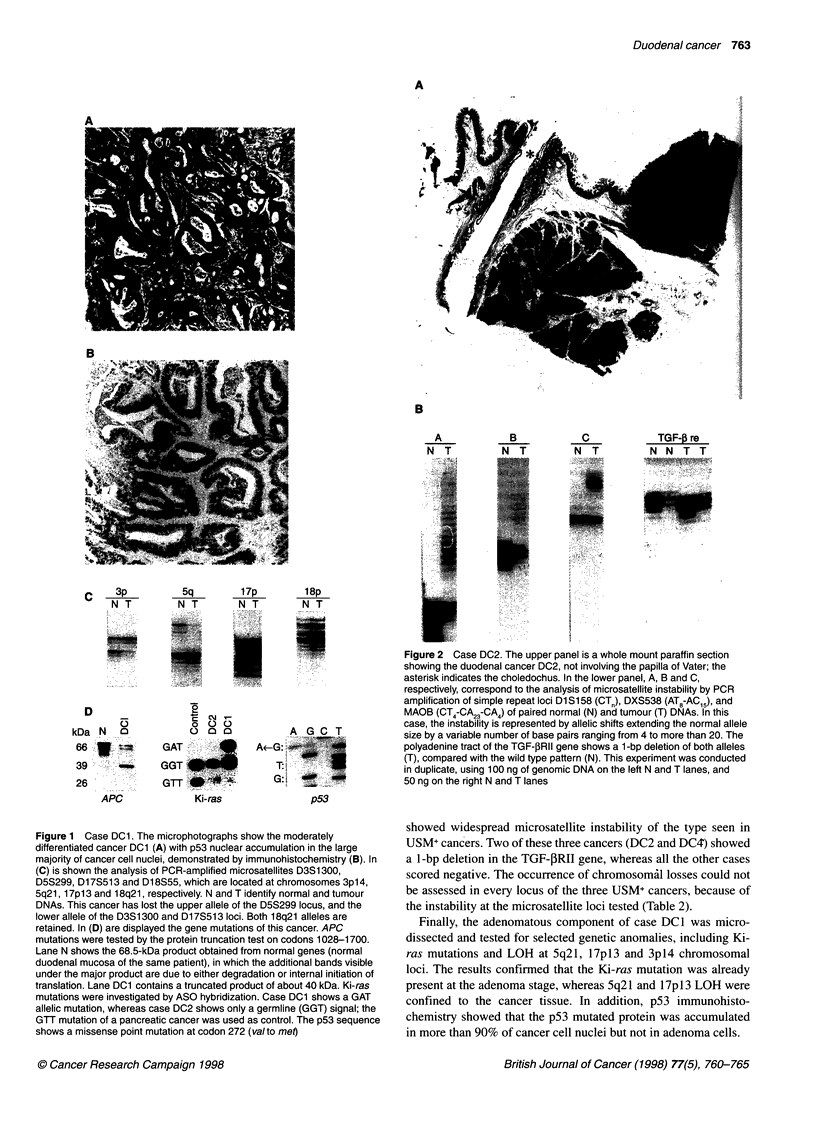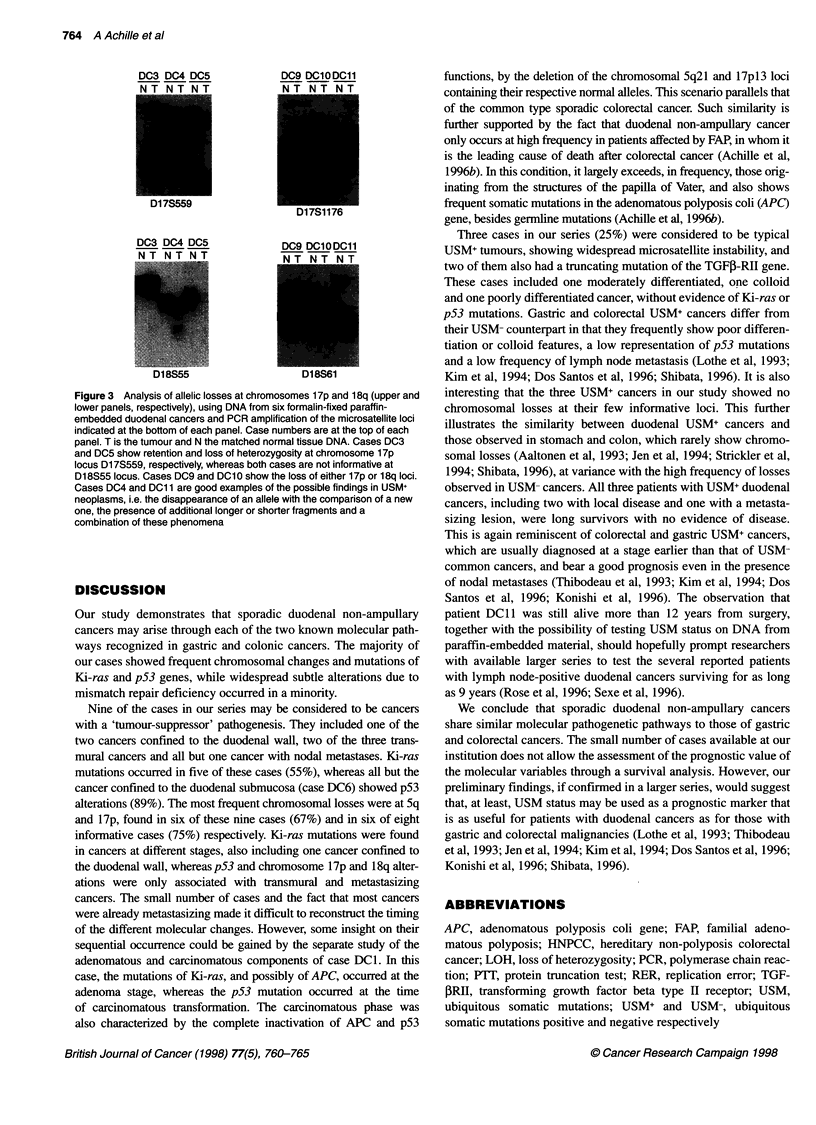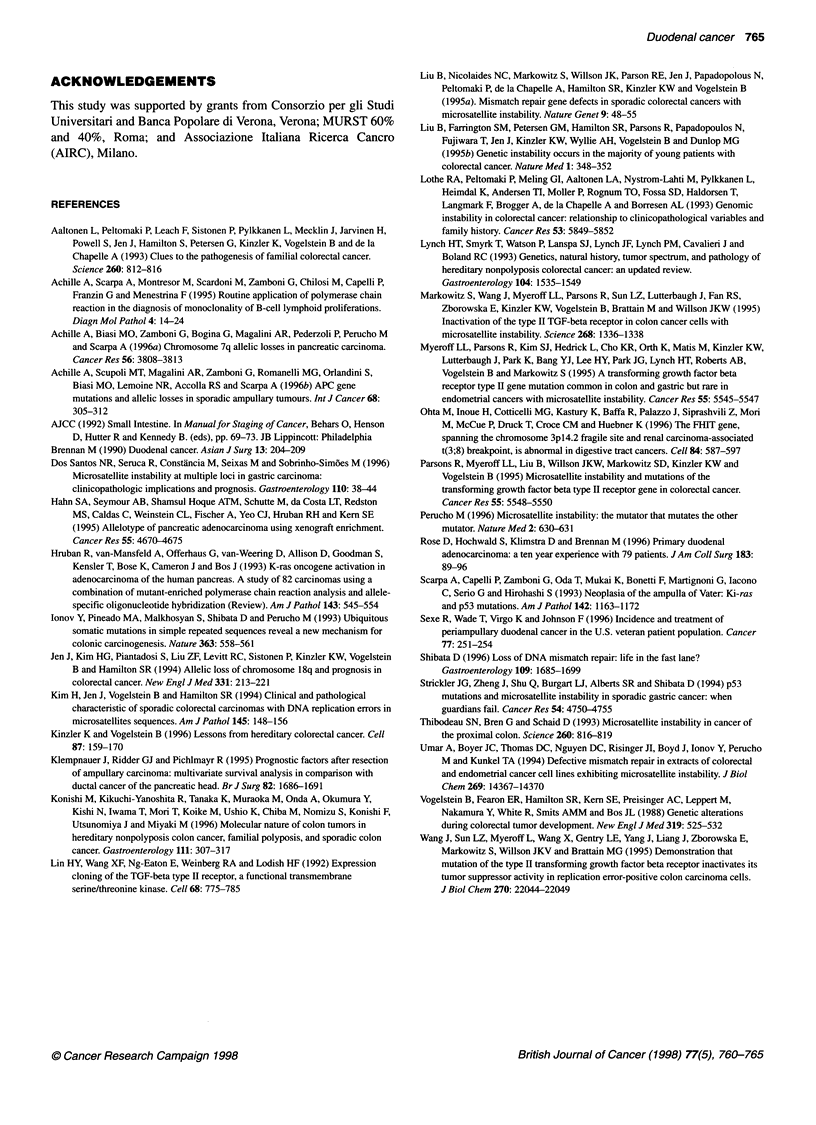# Molecular pathogenesis of sporadic duodenal cancer.

**DOI:** 10.1038/bjc.1998.124

**Published:** 1998-03

**Authors:** A. Achille, A. Baron, G. Zamboni, S. Orlandini, G. Bogina, C. Bassi, C. Iacono, A. Scarpa

**Affiliations:** Istituto di Anatomia Patologica, UniversitÃ di Verona, Italy.

## Abstract

**Images:**


British Journal of Cancer (1998) 77(5), 760–765
© 1998 Cancer Research Campaign

### Molecular pathogenesis of sporadic duodenal cancer

**A Achille[1], A Baron[1], G Zamboni[1], S Orlandini[1], G Bogina[1], C Bassi[2], C Iacono[2] and A Scarpa[1]**

[1]Istituto di Anatomia Patologica, Università di Verona, Strada Le Grazie 8, I-37134 Verona, Italy; [2]Dipartimento di Scienze Chirurgiche, Policlinico, I-37134, Verona, Italy

**Summary** Whether duodenal adenocarcinoma should be considered as a gastrointestinal or as a peripancreatic cancer is a matter of debate, as is the opportunity and type of treatment. We investigated 12 such cancers for the genetic anomalies involved in the pathogenesis of gastrointestinal malignancies, including (a) those occurring in common-type cancers – allelic losses at chromosomes 3p, 5q, 17p and 18q, and Ki-*ras* and *p53* alterations; and (b) those characteristic of mutator-phenotype cancers – microsatellite instability and *TGF-βRII* gene mutations. We found Ki-*ras* and *p53* mutations in five (42%) and eight cancers (67%), respectively; chromosome 3p, 5q, 17p and 18q allelic losses in two of nine (22%), six of ten (60%), six of nine (67%) and three of ten (30%) informative cancers, respectively. Finally, three cancers (25%) showed widespread microsatellite instability and two of them had a *TGF-βRII* gene mutation. Our data suggest that duodenal cancers may arise from either of the two known pathogenetic molecular pathways of gastric and colorectal cancers. The majority of our cases were highly aggressive cancers with frequent chromosomal changes and *p53* mutations as observed in the common-type gastrointestinal malignancies, while widespread subtle alterations characteristic of mutator-phenotype cancers occurred in a minority, which also showed a favourable long-term outcome.

**Keywords:** duodenal cancer; Ki-*ras*; APC; *p53*; microsatellite instability; *TGF-βRII*; loss of heterozygosity

Sporadic cancer of the duodenum not originating from the ampulla of Vater is a very rare disease. Yet it is the most common adenocarcinoma of the small intestine and kills 70% of affected patients, with a median survival of 20 months from diagnosis (Rose et al, 1996; Sexe et al, 1996). The natural history and outcome of this disease are poorly defined, because of its rarity and the inclusion of the few cases described in the literature within the category of periampullary cancers. The decision as to whether a duodenal adenocarcinoma should be viewed as a gastrointestinal cancer or as a peripancreatic cancer, with which the presentation is usually confused, is unclear (Brennan, 1990; Klempnauer et al, 1995; Rose et al, 1996). This is not a trivial question, as it also involves controversy on the opportunity and type of surgical and/or chemotherapeutic treatment (Brennan, 1990; Klempnauer et al, 1995; Rose et al, 1996). The study of duodenal cancers may also help to unravel the molecular pathogenesis of cancers arising in the periampullary area, including cancers of the papilla of Vater, of the biliary tract and of the pancreatic head.

Two molecular pathways are known to lead to gastric and colorectal cancer, termed the 'tumour suppressor' and the 'mutator' pathways (Perucho, 1996; Shibata, 1996). To the first belong the more common sporadic cancers, in which the pacemakers of tumour genesis and progression are the gross chromosomal changes and discrete mutations leading to the alteration of oncogenes and tumour suppressor genes, involved in the control of cell proliferation and death (Vogelstein et al, 1988; Kinzler and Vogelstein, 1996; Shibata, 1996). These malignancies are characterized by aneuploidy at the cytogenetic level and, at the molecular level, by allelic losses in an average of at least 25% of randomly chosen DNA sequences (Aaltonen et al, 1993). The prototypic hereditary cancer syndrome of this pathway is familial adenomatous polyposis (FAP) (Kinzler and Vogelstein, 1996; Perucho, 1996). The 'mutator pathway' has been recognized in a subset of sporadic gastric and colorectal, but not pancreatic, cancers (Ionov et al, 1993; Thibodeau et al, 1993; Strickler et al, 1994; Hahn et al, 1995; Konishi et al, 1996) and is a feature of cancers arising in hereditary non-polyposis colorectal cancer (HNPCC) (Lynch et al, 1993; Konishi et al, 1996). These cancers have been defined as $USM^+$ (ubiquitous somatic mutations positive), $RER^+$ (replication error positive) or as being of the mutator phenotype. They have defective DNA mismatch repair genes (mutator genes) (Umar et al, 1994; Liu et al, 1995*a*), causing the accumulation of hundreds of thousands of unrepaired mutations during cell replication cycles. Such a 'DNA phenotype', that is, the existence of widespread mutations throughout the genome, is easily detected by the analysis of simple repeat motifs of 1–4 bp (microsatellites), which are particularly prone to insertions or deletions (Liu et al, 1995*b*). In addition, $USM^+$ gastrointestinal cancers are usually euploid or near-diploid and rarely show chromosomal losses (Strickler et al, 1994; Kinzler and Vogelstein, 1996). In these cancers, the impaired function of DNA repair genes also causes the mutational activation or inactivation of functional genes (Shibata, 1996). In particular, the tumour growth factor-β type II receptor gene (*TGF-βRII*) is inactivated in almost all $USM^+$ gastric and colonic cancers, which escape, in this way, the TGF-β-mediated growth control (Markowitz et al, 1995; Myeroff et al, 1995; Parsons et al, 1995; Wang et al, 1995; Shibata, 1996).

We analysed paraffin-embedded samples from 12 duodenal cancers for the genetic anomalies involved in the genesis and progression of malignancy of gastrointestinal cancers. These included (a) those characteristic of tumour suppressor pathogenesis – alterations of Ki-*ras* gene and p53 protein; loss of heterozygosity

*Received 23 April 1997*
*Revised 23 April 1997*
*Accepted 18 July 1997*

*Correspondence to:* A Scarpa

**Table 1** Clinicopathological data for the duodenal cancers, listed according to the stage of the disease

| Case | Sex | Age (years) | Diagnosis | Size (cm) | Grade[a] | Stage[b] | Ki-*ras* mutation | p53 nuclear accumulation | Chromosomal loss[c] | | | | RER$^+$ phenotype | TGF-βRII mutation | Follow-up (months) | Outcome[d] |
|---|---|---|---|---|---|---|---|---|---|---|---|---|---|---|---|---|
| | | | | | | | | | 3p | 5q | 17p | 18q | | | | |
| DC 2 | M | 50 | Carcinoma | 1.5 | M | I | No | No | No | – | No | No | Yes | Yes | 42 | AW |
| DC 6 | F | 42 | Carcinoma | 2.7 | M | I | Yes | No | NI | No | No | No | No | No | 79 | AW |
| DC 3 | M | 54 | Carcinoma | 4 | M | II | Yes | Yes | No | Yes | No | No | No | No | 33 | AW |
| DC 4 | M | 42 | Carcinoma | 5.5 | Colloid | II | No | No | – | No | – | – | Yes | Yes | 25 | AW |
| DC 5 | F | 45 | Carcinoma | 2 | M | II | No | Yes | No | Yes | Yes | No | No | No | 66 | AW |
| DC 1 | M | 56 | Carcinoma | 2.5 | P | III | Yes | Yes | Yes | Yes | Yes | No | No | No | 12 | DOD |
| DC 7 | M | 67 | Carcinoma | 10 | P | III | No | Yes | No | Yes | NI | Yes | No | No | 13 | AD |
| DC 8 | F | 62 | Carcinoma | 7 | M | III | Yes | Yes | Yes | No | Yes | No | No | No | 15 | AD |
| DC 9 | F | 42 | Carcinoma | 6.5 | M | III | Yes | Yes | No | Yes | Yes | Yes | No | No | 16 | DOD |
| DC 10 | M | 50 | Carcinoma | 5 | P | III | No | Yes | No | No | Yes | Yes | No | No | 5 | AW |
| DC 11 | M | 52 | Carcinoma | 1 | P | III | No | No | – | – | – | – | Yes | No | 146 | AW |
| DC 12 | F | 55 | Carcinoma | 4 | M | III | No | Yes | No | Yes | Yes | No | No | No | 18 | DOD |

[a]M, moderately differentiated; P, poorly differentiated. [b]I, confined to the duodenal wall; II, transmural involvement; III, lymph node metastases. [c]NI, not informative; –, not evaluable for instability of all microsatellites analysed. [d]AW, alive and well; AD, alive with disease; DOD, died of disease.

(LOH) at chromosomal arms 5q21, 17p13, 18q21 and 3p14, which include the loci of *APC* (adenomatous polyposis coli), *p53*, *DCC* (deleted in colon cancer) and *FHIT* tumour-suppressor genes respectively (Vogelstein et al, 1988; Kinzler and Vogelstein, 1996; Ohta et al, 1996); and (b) those characteristic of the USM$^+$ cancers – microsatellite instability and truncating mutations in *TGF-βRII* receptor gene. In two cases, for which high-molecular-weight DNA from frozen samples was available, we could also characterize *APC* and *p53* gene mutations.

We report that sporadic cancers of duodenal non-ampullary origin may arise from either the tumour-suppressor or the mutator pathway, thus parallelling the molecular pathogenesis of gastric and colorectal cancers.

### MATERIALS AND METHODS

### Patients and tumours

Twelve cancers of unequivocal duodenal origin were retrieved from the files of the Department of Pathology of Verona University, Italy. They were selected from cancers of patients who underwent pancreaticoduodenectomy in the period 1985–1997. Ampullary and all periampullary cancers for which unequivocal duodenal origin could not be established were excluded from the study. The patients included seven men and five women, whose clinicopathological characteristics are reported in Table 1. All cancers but two (DC3 and DC5) presented as ulcerated masses. Staging was performed using the American Joint Committee on Cancer (AJCC) criteria for small intestinal malignancies (AJCC, 1992) (Table 1). Two cancers were confined to the duodenal wall: DC2 involved the muscularis propria and DC6 the duodenal submucosa. Three were transmural cancers (DC3, DC4 and DC5), with infiltration of the periduodenal fat and pancreas. The average number of lymph nodes isolated from the surgical specimens was 16 (range 4–33), and nodal metastases were present in seven cancers. All patients had a negative personal and familial history for cancer, including FAP.

In all cases, cancer cell-rich areas and matched normal mucosa were isolated from formalin-fixed paraffin-embedded blocks, and DNA was extracted as described (Achille et al, 1995). In cases DC1 and DC2, frozen samples were also available and were enriched to a neoplastic cellularity of more than 70% by cryostat dissection (Achille et al, 1996*a*). In these two cases, high-molecular-weight DNA was prepared by standard methods from 80 6-μm cryostatic sections, the cell composition of the sample being ascertained every 20 sections, and normal DNA was obtained from frozen strips of normal duodenal mucosa of each patient.

### Molecular analysis

All cases were studied for mutations of Ki-*ras* and *TGF-βRII* genes, allelic losses at chromosomes 3p, 5q, 17p and 18q using polymerase chain reaction (PCR)-based methods, as well as for p53 protein nuclear accumulation using immunohistochemistry. Mutations of *APC* and *p53* genes were only analysed in the two cases with available high-molecular-weight DNA from frozen tissues, because of the inadequacy of degraded DNA from paraffin-embedded tissues for such analyses.

Mutations of Ki-*ras* codon 12 were screened by mutant-enriched PCR and characterized by allele-specific oligonucleotide hybridization (ASO), according to Hruban et al (1993). Immunohistochemistry for p53 protein was performed on paraffin sections, using pAb1801 monoclonal antibody (Scarpa et al, 1993). Truncating mutations of *APC* gene were searched using the APC protein truncation test (PTT) analysis of codons 654–1700, as previously described in detail (Achille et al, 1996*b*). Mutations of the *p53* gene were investigated using single-strand conformation polymorphism and direct DNA sequencing of PCR-amplified DNA fragments (Scarpa et al, 1993).

Microsatellite instability was assessed through PCR amplification of four loci, including D1S158, DXS538, MAOB and APΔ3. LOH at specific chromosomal loci was examined by PCR amplification of ten microsatellites. These included D5S82 and D5S299 for chromosome 5q21; D17S513, D17S1176 and D17S559 for chromosome 17p13; D18S65, D18S61 and D18S55 for chromosome 18q21; D3S1234 and D3S1300 for chromosome 3p14. All appropriate primers for amplification of microsatellites were purchased from the MapPairs collection (Research Genetics, Huntsville, AL, USA). They were used at the annealing temperature indicated by the manufacturer when using high-quality DNA from frozen tissues and at 5°C lower when using DNA from paraffin-embedded tissues. The PCR reaction (10 μl) and product detection for microsatellite instability and LOH studies were performed as previously described in detail for high-quality DNAs

© Cancer Research Campaign 1998

**Table 2** Detailed results of loss of heterozygosity by microsatellite analysis[a]

| Case | Chromosome 3p14 | | Chromosome 5q21 | | Chromosome 17p13 | | | Chromosome 18q21 | | |
|---|---|---|---|---|---|---|---|---|---|---|
| | D3S1234 | D3S1300 | D5S82 | D5S299 | D17S513 | D17S1176 | D17S559 | D18S65 | D18S61 | D18S55 |
| DC 2 | – | No | – | – | [b] | [b] | [b] | – | – | No |
| DC 6 | NI | Not ampl. | No | No | No | No | NI | NI | No | No |
| DC 3 | No | NI | Loss | NI | NI | No | No | No | No | n.i. |
| DC 4 | Not ampl. | – | NI | No | Not ampl. | – | – | Not ampl. | – | – |
| DC 5 | No | No | NI | Loss | NI | Loss | Loss | No | NI | NI |
| DC 1 | NI | Loss | NI | Loss | Loss | NI | Loss | No | NI | No |
| DC 7 | No | Not ampl. | Loss | Loss | Not ampl. | NI | NI | NI | Loss | Loss |
| DC 8 | Loss | Loss | NI | No | Loss | Loss | Loss | No | NI | Not ampl. |
| DC 9 | NI | No | Loss | Loss | NI | Loss | Loss | Loss | Loss | Not ampl. |
| DC 10 | No | NI | No | NI | Loss | Loss | NI | NI | Loss | NI |
| DC 11 | – | – | – | Not ampl. | – | Not ampl. | – | – | Not ampl. | – |
| DC 12 | No | No | Loss | NI | Loss | Not ampl. | NI | No | NI | No |

[a]NI, not informative; –, not evaluable for instability of all microsatellites analysed; not ampl. stands for no PCR amplification of either normal or tumour DNA.
[b]The absence of 17p loss was demonstrated by Southern blot (see Results). Note that, of the three USM+ cancers, DC2 had two stable and informative loci, DC4 had two stable but only one informative loci and for DC11 all locus were unstable.

(Achille et al, 1996*a* and *b*), with a modification for DNA from paraffin-embedded tissues, consisting of the addition of five PCR cycles. Microsatellite instability was defined when a shift and/or gain of electrophoretic bands was detected (Kim et al, 1994), whereas LOH was characterized by loss of the bands representing one allele (Achille et al, 1996*a*).

The TGF-βRII gene was analysed by PCR amplification of the 73-bp (nucleotides 665–737) which contains the mutational hotspot represented by a 10-bp polyadenine tract (codons 125–128) (Markowitz et al, 1995; Myeroff et al, 1995; Parsons et al, 1995). Genomic DNA (50 and 100 ng) was amplified in duplicate experiments using forward [γ-$^{32}$P]ATP 5′-end-labelled primer TA10-F1 (5′-CTTTATTCTGGAAGATGCTGC-3′) and reverse primer TA10-R1 (5′-GAAGAAAGTCTCACCAGG-3′) (Myeroff et al, 1995). PCR for both high-quality and partly degraded DNAs consisted of 30 cycles of 95°C for 30 s, 55°C for 60 s and 72°C for 60 s. The PCR products were electrophoresed for 1 h at 60 W in 6% polyacrylamide (5% cross-linker, 0.2 mm thick) gel containing 8 M urea and were visualized by autoradiography. The standard in each assay was established by amplifying the wild-type TGF-βRII gene from plasmid H2-3FF (Lin et al, 1992), kindly provided by Professor RA Weinberg (Whithead Institute, MIT, Cambridge, MA, USA).

### RESULTS

The results of the molecular studies are summarized in Table 1, and details of chromosomal allelic losses are given in Table 2. We first analysed the two cancers for which high-quality DNA from frozen samples was available (DC1 and DC2). After testing the suitability for genetic analysis of the partly degraded DNA from formalin-fixed paraffin-embedded samples, as processed at our institution, we extended the study to the additional ten cases of our series.

### Molecular analysis of cancers DC1 and DC2

Cancer DC1 showed mutations in *APC*, Ki-*ras* and *p53* genes, together with allelic losses at chromosomal loci 5q21, 17p13 and 3p14 (Figure 1). Chromosome 18q21 alleles were retained, and neither microsatellite instability nor insertions or deletions in the *TGF-βRII* gene were found.

Cancer DC2 showed instability at all four microsatellites used for detection of this anomaly (Figure 2), and also in the majority of the microsatellites (eight of ten) used for LOH analyses. Therefore, it was classified as a USM+ neoplasm. The TGF-βRII gene of this cancer had a 1-bp deletion in both alleles within the polyadenine tract. Such biallelic mutation predicts an inactive truncated receptor protein of 161 amino acids (Myeroff et al, 1995). This case had neither *APC*, nor Ki-*ras* or *p53* mutations. The two microsatellites not showing instability, D3S1300 and D18S55, were both informative and showed no allelic loss. The absence of allelic losses at the 17p13 locus was demonstrated by Southern blot analysis of *Msp*I-digested DNA hybridized to the pYNZ22 probe (American Type Culture Collection, Rockville, MD, USA) (data not shown).

### Adequacy of paraffin-embedded samples and summary of molecular anomalies

We assessed the adequacy of formalin-fixed and paraffin-embedded tissue from our institution by testing the DNA extracted from paraffin blocks of cancers DC1 and DC2 for Ki-*ras* and TGF-βRII mutations, microsatellite instability and LOH at chromosomes 17p and 18q (Figure 3). The results perfectly matched those obtained with DNA from frozen tissues. We then extended the study to the remaining ten cases, whose partly degraded DNA could be successfully tested for all the molecular anomalies analysed in cases DC1 and DC2, with the exception of *APC* and *p53* gene mutations. However, the detection of p53 protein accumulation in cell nuclei by immunohistochemistry serves as a surrogate for *p53* gene mutations in gastrointestinal cancers (Kim et al, 1994; Achille et al, 1996*b*).

In summary, *Ki-ras* codon 12 mutations were detected in 5 of the 12 cancers (42%). They were GGT to GAT in four instances and GGT to GCT in one. Abnormal accumulation of p53 protein was found in eight cancers (67%), all showing immunostaining in the vast majority of cancer cell nuclei. Six of these eight cases had a complete loss of p53 function, as suggested by the concomitant loss of the normal p53 allele on chromosome 17p. Allelic losses at chromosomes 3p, 5q, 17p and 18q were found in two of nine (22%), six of ten (60%), six of nine (67%) and three of ten (30%) informative cancers, respectively. Three of the 12 cancers (25%)

 © Cancer Research Campaign 1998

**Figure 1** Case DC1. The microphotographs show the moderately differentiated cancer DC1 (**A**) with p53 nuclear accumulation in the large majority of cancer cell nuclei, demonstrated by immunohistochemistry (**B**). In (**C**) is shown the analysis of PCR-amplified microsatellites D3S1300, D5S299, D17S513 and D18S55, which are located at chromosomes 3p14, 5q21, 17p13 and 18q21, respectively. N and T identify normal and tumour DNAs. This cancer has lost the upper allele of the D5S299 locus, and the lower allele of the D3S1300 and D17S513 loci. Both 18q21 alleles are retained. In (**D**) are displayed the gene mutations of this cancer. *APC* mutations were tested by the protein truncation test on codons 1028–1700. Lane N shows the 68.5-kDa product obtained from normal genes (normal duodenal mucosa of the same patient), in which the additional bands visible under the major product are due to either degradation or internal initiation of translation. Lane DC1 contains a truncated product of about 40 kDa. Ki-*ras* mutations were investigated by ASO hybridization. Case DC1 shows a GAT allelic mutation, whereas case DC2 shows only a germline (GGT) signal; the GTT mutation of a pancreatic cancer was used as control. The p53 sequence shows a missense point mutation at codon 272 (*val* to *met*)

**Figure 2** Case DC2. The upper panel is a whole mount paraffin section showing the duodenal cancer DC2, not involving the papilla of Vater; the asterisk indicates the choledochus. In the lower panel, A, B and C, respectively, correspond to the analysis of microsatellite instability by PCR amplification of simple repeat loci D1S158 ($CT_n$), DXS538 ($AT_8$-$AC_{15}$), and MAOB ($CT_4$-$CA_{23}$-$CA_4$) of paired normal (N) and tumour (T) DNAs. In this case, the instability is represented by allelic shifts extending the normal allele size by a variable number of base pairs ranging from 4 to more than 20. The polyadenine tract of the TGF-βRII gene shows a 1-bp deletion of both alleles (T), compared with the wild type pattern (N). This experiment was conducted in duplicate, using 100 ng of genomic DNA on the left N and T lanes, and 50 ng on the right N and T lanes showed widespread microsatellite instability of the type seen in USM+ cancers. Two of these three cancers (DC2 and DC4) showed a 1-bp deletion in the TGF-βRII gene, whereas all the other cases scored negative. The occurrence of chromosomal losses could not be assessed in every locus of the three USM+ cancers, because of the instability at the microsatellite loci tested (Table 2).

Finally, the adenomatous component of case DC1 was microdissected and tested for selected genetic anomalies, including Ki-*ras* mutations and LOH at 5q21, 17p13 and 3p14 chromosomal loci. The results confirmed that the Ki-*ras* mutation was already present at the adenoma stage, whereas 5q21 and 17p13 LOH were confined to the cancer tissue. In addition, p53 immunohistochemistry showed that the p53 mutated protein was accumulated in more than 90% of cancer cell nuclei but not in adenoma cells.

© Cancer Research Campaign 1998 

**Figure 3** Analysis of allelic losses at chromosomes 17p and 18q (upper and lower panels, respectively), using DNA from six formalin-fixed paraffin-embedded duodenal cancers and PCR amplification of the microsatellite loci indicated at the bottom of each panel. Case numbers are at the top of each panel. T is the tumour and N the matched normal tissue DNA. Cases DC3 and DC5 show retention and loss of heterozygosity at chromosome 17p locus D17S559, respectively, whereas both cases are not informative at D18S55 locus. Cases DC9 and DC10 show the loss of either 17p or 18q loci. Cases DC4 and DC11 are good examples of the possible findings in USM+ neoplasms, i.e. the disappearance of an allele with the comparison of a new one, the presence of additional longer or shorter fragments and a combination of these phenomena

### DISCUSSION

Our study demonstrates that sporadic duodenal non-ampullary cancers may arise through each of the two known molecular pathways recognized in gastric and colonic cancers. The majority of our cases showed frequent chromosomal changes and mutations of Ki-*ras* and *p53* genes, while widespread subtle alterations due to mismatch repair deficiency occurred in a minority.

Nine of the cases in our series may be considered to be cancers with a 'tumour-suppressor' pathogenesis. They included one of the two cancers confined to the duodenal wall, two of the three transmural cancers and all but one cancer with nodal metastases. Ki-*ras* mutations occurred in five of these cases (55%), whereas all but the cancer confined to the duodenal submucosa (case DC6) showed p53 alterations (89%). The most frequent chromosomal losses were at 5q and 17p, found in six of these nine cases (67%) and in six of eight informative cases (75%) respectively. Ki-*ras* mutations were found in cancers at different stages, also including one cancer confined to the duodenal wall, whereas *p53* and chromosome 17p and 18q alterations were only associated with transmural and metastasizing cancers. The small number of cases and the fact that most cancers were already metastasizing made it difficult to reconstruct the timing of the different molecular changes. However, some insight on their sequential occurrence could be gained by the separate study of the adenomatous and carcinomatous components of case DC1. In this case, the mutations of Ki-*ras*, and possibly of *APC*, occurred at the adenoma stage, whereas the *p53* mutation occurred at the time of carcinomatous transformation. The carcinomatous phase was also characterized by the complete inactivation of APC and p53 functions, by the deletion of the chromosomal 5q21 and 17p13 loci containing their respective normal alleles. This scenario parallels that of the common type sporadic colorectal cancer. Such similarity is further supported by the fact that duodenal non-ampullary cancer only occurs at high frequency in patients affected by FAP, in whom it is the leading cause of death after colorectal cancer (Achille et al, 1996*b*). In this condition, it largely exceeds, in frequency, those originating from the structures of the papilla of Vater, and also shows frequent somatic mutations in the adenomatous polyposis coli (*APC*) gene, besides germline mutations (Achille et al, 1996*b*).

Three cases in our series (25%) were considered to be typical USM$^+$ tumours, showing widespread microsatellite instability, and two of them also had a truncating mutation of the TGFβ-RII gene. These cases included one moderately differentiated, one colloid and one poorly differentiated cancer, without evidence of Ki-*ras* or *p53* mutations. Gastric and colorectal USM$^+$ cancers differ from their USM$^-$ counterpart in that they frequently show poor differentiation or colloid features, a low representation of *p53* mutations and a low frequency of lymph node metastasis (Lothe et al, 1993; Kim et al, 1994; Dos Santos et al, 1996; Shibata, 1996). It is also interesting that the three USM$^+$ cancers in our study showed no chromosomal losses at their few informative loci. This further illustrates the similarity between duodenal USM$^+$ cancers and those observed in stomach and colon, which rarely show chromosomal losses (Aaltonen et al, 1993; Jen et al, 1994; Strickler et al, 1994; Shibata, 1996), at variance with the high frequency of losses observed in USM$^-$ cancers. All three patients with USM$^+$ duodenal cancers, including two with local disease and one with a metastasizing lesion, were long survivors with no evidence of disease. This is again reminiscent of colorectal and gastric USM$^+$ cancers, which are usually diagnosed at a stage earlier than that of USM$^-$ common cancers, and bear a good prognosis even in the presence of nodal metastases (Thibodeau et al, 1993; Kim et al, 1994; Dos Santos et al, 1996; Konishi et al, 1996). The observation that patient DC11 was still alive more than 12 years from surgery, together with the possibility of testing USM status on DNA from paraffin-embedded material, should hopefully prompt researchers with available larger series to test the several reported patients with lymph node-positive duodenal cancers surviving for as long as 9 years (Rose et al, 1996; Sexe et al, 1996).

We conclude that sporadic duodenal non-ampullary cancers share similar molecular pathogenetic pathways to those of gastric and colorectal cancers. The small number of cases available at our institution does not allow the assessment of the prognostic value of the molecular variables through a survival analysis. However, our preliminary findings, if confirmed in a larger series, would suggest that, at least, USM status may be used as a prognostic marker that is as useful for patients with duodenal cancers as for those with gastric and colorectal malignancies (Lothe et al, 1993; Thibodeau et al, 1993; Jen et al, 1994; Kim et al, 1994; Dos Santos et al, 1996; Konishi et al, 1996; Shibata, 1996).

### ABBREVIATIONS

*APC*, adenomatous polyposis coli gene; FAP, familial adenomatous polyposis; HNPCC, hereditary non-polyposis colorectal cancer; LOH, loss of heterozygosity; PCR, polymerase chain reaction; PTT, protein truncation test; RER, replication error; TGF-βRII, transforming growth factor beta type II receptor; USM, ubiquitous somatic mutations; USM$^+$ and USM$^-$, ubiquitous somatic mutations positive and negative respectively

 © Cancer Research Campaign 1998

### ACKNOWLEDGEMENTS

This study was supported by grants from Consorzio per gli Studi Universitari and Banca Popolare di Verona, Verona; MURST 60% and 40%, Roma; and Associazione Italiana Ricerca Cancro (AIRC), Milano.

### REFERENCES

Aaltonen L, Peltomaki P, Leach F, Sistonen P, Pylkkanen L, Mecklin J, Jarvinen H, Powell S, Jen J, Hamilton S, Petersen G, Kinzler K, Vogelstein B and de la Chapelle A (1993) Clues to the pathogenesis of familial colorectal cancer. *Science* **260**: 812–816

Achille A, Scarpa A, Montresor M, Scardoni M, Zamboni G, Chilosi M, Capelli P, Franzin G and Menestrina F (1995) Routine application of polymerase chain reaction in the diagnosis of monoclonality of B-cell lymphoid proliferations. *Diagn Mol Pathol* **4**: 14–24

Achille A, Biasi MO, Zamboni G, Bogina G, Magalini AR, Pederzoli P, Perucho M and Scarpa A (1996*a*) Chromosome 7q allelic losses in pancreatic carcinoma. *Cancer Res* **56**: 3808–3813

Achille A, Scupoli MT, Magalini AR, Zamboni G, Romanelli MG, Orlandini S, Biasi MO, Lemoine NR, Accolla RS and Scarpa A (1996*b*) APC gene mutations and allelic losses in sporadic ampullary tumours. *Int J Cancer* **68**: 305-312

AJCC (1992) Small Intestine. In *Manual for Staging of Cancer*, Behars O, Henson D, Hutter R and Kennedy B. (eds), pp. 69–73. JB Lippincott: Philadelphia

Brennan M (1990) Duodenal cancer. *Asian J Surg* **13**: 204–209

Dos Santos NR, Seruca R, Constância M, Seixas M and Sobrinho-Simões M (1996) Microsatellite instability at multiple loci in gastric carcinoma: clinicopathologic implications and prognosis. *Gastroenterology* **110**: 38–44

Hahn SA, Seymour AB, Shamsul Hoque ATM, Schutte M, da Costa LT, Redston MS, Caldas C, Weinstein CL, Fischer A, Yeo CJ, Hruban RH and Kern SE (1995) Allelotype of pancreatic adenocarcinoma using xenograft enrichment. *Cancer Res* **55**: 4670–4675

Hruban R, van-Mansfeld A, Offerhaus G, van-Weering D, Allison D, Goodman S, Kensler T, Bose K, Cameron J and Bos J (1993) K-ras oncogene activation in adenocarcinoma of the human pancreas. A study of 82 carcinomas using a combination of mutant-enriched polymerase chain reaction analysis and allele-specific oligonucleotide hybridization (Review). *Am J Pathol* **143**: 545–554

Ionov Y, Pineado MA, Malkhosyan S, Shibata D and Perucho M (1993) Ubiquitous somatic mutations in simple repeated sequences reveal a new mechanism for colonic carcinogenesis. *Nature* **363**: 558–561

Jen J, Kim HG, Piantadosi S, Liu ZF, Levitt RC, Sistonen P, Kinzler KW, Vogelstein B and Hamilton SR (1994) Allelic loss of chromosome 18q and prognosis in colorectal cancer. *New Engl J Med* **331**: 213–221

Kim H, Jen J, Vogelstein B and Hamilton SR (1994) Clinical and pathological characteristic of sporadic colorectal carcinomas with DNA replication errors in microsatellites sequences. *Am J Pathol* **145**: 148–156

Kinzler K and Vogelstein B (1996) Lessons from hereditary colorectal cancer. *Cell* **87**: 159–170

Klempnauer J, Ridder GJ and Pichlmayr R (1995) Prognostic factors after resection of ampullary carcinoma: multivariate survival analysis in comparison with ductal cancer of the pancreatic head. *Br J Surg* **82**: 1686–1691

Konishi M, Kikuchi-Yanoshita R, Tanaka K, Muraoka M, Onda A, Okumura Y, Kishi N, Iwama T, Mori T, Koike M, Ushio K, Chiba M, Nomizu S, Konishi F, Utsunomiya J and Miyaki M (1996) Molecular nature of colon tumors in hereditary nonpolyposis colon cancer, familial polyposis, and sporadic colon cancer. *Gastroenterology* **111**: 307–317

Lin HY, Wang XF, Ng-Eaton E, Weinberg RA and Lodish HF (1992) Expression cloning of the TGF-beta type II receptor, a functional transmembrane serine/threonine kinase. *Cell* **68**: 775–785

Liu B, Nicolaides NC, Markowitz S, Willson JK, Parson RE, Jen J, Papadopolous N, Peltomaki P, de la Chapelle A, Hamilton SR, Kinzler KW and Vogelstein B (1995*a*). Mismatch repair gene defects in sporadic colorectal cancers with microsatellite instability. *Nature Genet* **9**: 48–55

Liu B, Farrington SM, Petersen GM, Hamilton SR, Parsons R, Papadopoulos N, Fujiwara T, Jen J, Kinzler KW, Wyllie AH, Vogelstein B and Dunlop MG (1995*b*) Genetic instability occurs in the majority of young patients with colorectal cancer. *Nature Med* **1**: 348–352

Lothe RA, Peltomaki P, Meling GI, Aaltonen LA, Nystrom-Lahti M, Pylkkanen L, Heimdal K, Andersen TI, Moller P, Rognum TO, Fossa SD, Haldorsen T, Langmark F, Brogger A, de la Chapelle A and Borresen AL (1993) Genomic instability in colorectal cancer: relationship to clinicopathological variables and family history. *Cancer Res* **53**: 5849–5852

Lynch HT, Smyrk T, Watson P, Lanspa SJ, Lynch JF, Lynch PM, Cavalieri J and Boland RC (1993) Genetics, natural history, tumor spectrum, and pathology of hereditary nonpolyposis colorectal cancer: an updated review. *Gastroenterology* **104**: 1535–1549

Markowitz S, Wang J, Myeroff LL, Parsons R, Sun LZ, Lutterbaugh J, Fan RS, Zborowska E, Kinzler KW, Vogelstein B, Brattain M and Willson JKW (1995) Inactivation of the type II TGF-beta receptor in colon cancer cells with microsatellite instability. *Science* **268**: 1336–1338

Myeroff LL, Parsons R, Kim SJ, Hedrick L, Cho KR, Orth K, Matis M, Kinzler KW, Lutterbaugh J, Park K, Bang YJ, Lee HY, Park JG, Lynch HT, Roberts AB, Vogelstein B and Markowitz S (1995) A transforming growth factor beta receptor type II gene mutation common in colon and gastric but rare in endometrial cancers with microsatellite instability. *Cancer Res* **55**: 5545–5547

Ohta M, Inoue H, Cotticelli MG, Kastury K, Baffa R, Palazzo J, Siprashvili Z, Mori M, McCue P, Druck T, Croce CM and Huebner K (1996) The FHIT gene, spanning the chromosome 3p14.2 fragile site and renal carcinoma-associated t(3;8) breakpoint, is abnormal in digestive tract cancers. *Cell* **84**: 587–597

Parsons R, Myeroff LL, Liu B, Willson JKW, Markowitz SD, Kinzler KW and Vogelstein B (1995) Microsatellite instability and mutations of the transforming growth factor beta type II receptor gene in colorectal cancer. *Cancer Res* **55**: 5548–5550

Perucho M (1996) Microsatellite instability: the mutator that mutates the other mutator. *Nature Med* **2**: 630–631

Rose D, Hochwald S, Klimstra D and Brennan M (1996) Primary duodenal adenocarcinoma: a ten year experience with 79 patients. *J Am Coll Surg* **183**: 89–96

Scarpa A, Capelli P, Zamboni G, Oda T, Mukai K, Bonetti F, Martignoni G, Iacono C, Serio G and Hirohashi S (1993) Neoplasia of the ampulla of Vater: Ki-*ras* and p53 mutations. *Am J Pathol* **142**: 1163–1172

Sexe R, Wade T, Virgo K and Johnson F (1996) Incidence and treatment of periampullary duodenal cancer in the U.S. veteran patient population. *Cancer* **77**: 251–254

Shibata D (1996) Loss of DNA mismatch repair: life in the fast lane? *Gastroenterology* **109**: 1685–1699

Strickler JG, Zheng J, Shu Q, Burgart LJ, Alberts SR and Shibata D (1994) p53 mutations and microsatellite instability in sporadic gastric cancer: when guardians fail. *Cancer Res* **54**: 4750–4755

Thibodeau SN, Bren G and Schaid D (1993) Microsatellite instability in cancer of the proximal colon. *Science* **260**: 816–819

Umar A, Boyer JC, Thomas DC, Nguyen DC, Risinger JI, Boyd J, Ionov Y, Perucho M and Kunkel TA (1994) Defective mismatch repair in extracts of colorectal and endometrial cancer cell lines exhibiting microsatellite instability. *J Biol Chem* **269**: 14367–14370

Vogelstein B, Fearon ER, Hamilton SR, Kern SE, Preisinger AC, Leppert M, Nakamura Y, White R, Smits AMM and Bos JL (1988) Genetic alterations during colorectal tumor development. *New Engl J Med* **319**: 525–532

Wang J, Sun LZ, Myeroff L, Wang X, Gentry LE, Yang J, Liang J, Zborowska E, Markowitz S, Willson JKV and Brattain MG (1995) Demonstration that mutation of the type II transforming growth factor beta receptor inactivates its tumor suppressor activity in replication error-positive colon carcinoma cells. *J Biol Chem* **270**: 22044–22049

© Cancer Research Campaign 1998